# Responses of Airway Epithelium to Environmental Injury: Role in the Induction Phase of Childhood Asthma

**DOI:** 10.1155/2011/257017

**Published:** 2011-11-01

**Authors:** Rakesh K. Kumar, Jessica S. Siegle, Gerard E. Kaiko, Cristan Herbert, Joerg E. Mattes, Paul S. Foster

**Affiliations:** ^1^Inflammation and Infection Research Centre, School of Medial Sciences, University of New South Wales, Sydney, NSW 2052, Australia; ^2^Centre for Asthma and Respiratory Disease, School of Biomedical Sciences, University of Newcastle and Hunter Medical Research Institute, Callaghan, NSW 2300, Australia

## Abstract

The pathogenesis of allergic asthma in childhood remains poorly understood. Environmental factors which appear to contribute to allergic sensitisation, with development of a Th2-biased immunological response in genetically predisposed individuals, include wheezing lower respiratory viral infections in early life and exposure to airborne environmental pollutants. These may activate pattern recognition receptors and/or cause oxidant injury to airway epithelial cells (AECs). In turn, this may promote Th2 polarisation via a “final common pathway” involving interaction between AEC, dendritic cells, and CD4+ T lymphocytes. Potentially important cytokines produced by AEC include thymic stromal lymphopoietin and interleukin-25. Their role is supported by in vitro studies using human AEC, as well as by experiments in animal models. To date, however, few investigations have employed models of the induction phase of childhood asthma. Further research may help to identify interventions that could reduce the risk of allergic asthma.

## 1. Introduction

Asthma is one of the most common chronic diseases affecting children, especially in economically developed nations. For example, in Australia the prevalence of current asthma in children aged 0–15 years is approximately 11% [1]. Childhood asthma is strongly linked to atopy, which in turn is characteristically associated with a Th2-biased immunological response [2–4]. While this relationship is well documented, the pathogenesis of childhood asthma remains largely unexplained.

However, it is clear that both genetic predisposition and a variety of environmental factors contribute to the development of allergic asthma [4]. Notable among the environmental factors that appear to be crucial in the induction of disease is respiratory viral infection, in particular with rhinovirus (RV) or respiratory syncytial virus (RSV). The association between childhood infections and asthma is complex, because at least in some settings, repeated early-life exposure to infectious agents may reduce the likelihood of developing allergic diseases [5]. Despite this, epidemiological studies strongly suggest that lower respiratory viral infections associated with wheezing, occurring within a critical period of development in early childhood, play an important role in the subsequent development of asthma in children who are repeatedly exposed to inhaled allergens [6–10].

Another clearly defined risk factor for childhood allergic asthma is early-life exposure to airborne environmental irritants. The importance of exposure to environmental tobacco smoke is well established [11, 12]. More recently, a number of large population-based studies, including prospective cohort studies, have clearly defined the increased risk of development of asthma in children exposed to traffic-related particulate pollutants [13–15]. The adverse respiratory effects of such pollutants, especially diesel exhaust particulates (DEPs), are now recognised as a significant public health problem [16]. 

Somewhat more contentious is the association between the use of paracetamol (acetaminophen) in infancy or childhood and the subsequent development of asthma [17, 18]. The evidence for an increased risk of asthma following exposures to other environmental chemicals is much less convincing [19].

Fundamental questions remain unanswered about the underlying mechanisms by which environmental factors promote the development of childhood asthma. In particular, if atopy and a Th2-biased immunological response are indeed precursors to the development of childhood asthma, a key issue is how does injury by environmental factors drive an allergic response? 

A possible “final common pathway,” for which there is now growing support, is based on the interaction between airway epithelial cells, dendritic cells, and CD4+ T-lymphocytes. 

## 2. Driving a Th2-Biased Immunological Response

**Figure 1:**
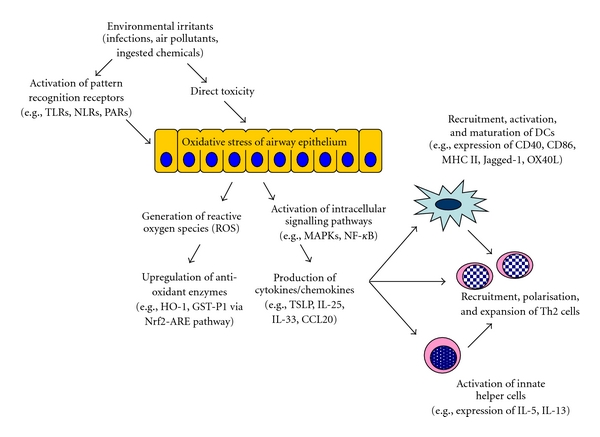
Environmental irritants may activate various pattern recognition receptors on AEC or may be directly toxic to the cells. Exposure to such irritants frequently causes oxidant injury to AEC, leading to generation of reactive oxygen species and upregulation of antioxidant enzyme systems. In parallel, intracellular signalling pathways are activated, triggering production of cytokines that can recruit and stimulate DC, upregulate their expression of costimulatory molecules and promote Th2 polarisation of the CD4+ T-cell response. These cytokines may also activate various populations of innate helper cells, leading to expansion of the Th2 cell population and helping to drive the allergic inflammatory response.

Dendritic cells (DCs) have long been recognised as playing a crucial role in the induction of Th2 polarisation during an immunological response [20]. As is increasingly being understood, the development of allergic immunological responses may be determined by innate host defence responses after initial exposure to pathogens, allergens, or other irritants [21–23]. These lead to local generation of cytokines that stimulate DC, with effects including upregulation of the expression of costimulatory molecules such as CD40, CD80, CD86, Jagged-1, and OX40L, as well as the production of various chemokines (Figure 1) [24–27]. Such molecules collectively function as “instructive” signals that drive initial Th2 polarisation. The subsequent maintenance of the Th2 bias of the CD4+ T cells may be dependent on epigenetic changes [28, 29], which is now a focus of considerable interest in the study of the pathogenesis of asthma [30, 31].

Key factors that may activate and/or drive the maturation of conventional or myeloid DC, to promote Th2-biased differentiation of CD4+ T-lymphocytes, include the cytokines granulocyte-macrophage colony-stimulating factor (GM-CSF), thymic stromal lymphopoietin (TSLP), interleukin (IL)-25 and IL-33 [32]. Because airway epithelial cells (AEC) can secrete GM-CSF, TSLP, IL-25, and IL-33 in response to injury, as well as chemoattractants for DC such as CCL20, the airway epithelium appears likely to play a critical role in promoting recruitment/survival of DC and Th2 polarisation of the immune response (Figure 1) [33–35].

### 2.1. Role of TSLP

Accumulating evidence indicates that TSLP activates DC to prime CD4+ T cells for inflammatory Th2 differentiation [36]. In the context of the induction of childhood asthma, a role for AEC-derived TSLP in the induction phase is strongly supported by in vitro studies. Following exposure to DEP in vitro, human AEC generated reactive oxygen species (ROS) and secreted TSLP, which caused DC precursors to enhance expression of OX40L and Jagged-1, which in turn promoted upregulation of Th2 responses [37, 38]. Similarly, AEC exposed to double-stranded RNA or infected with RV or RSV in vitro exhibited marked upregulation of expression of TSLP [39, 40]. In vivo, transgenic overexpression of TSLP in the lungs promoted an antigen-driven allergic inflammatory response [41]. Injury by proteases has also been shown to elicit generation of ROS by epithelial cells, leading to oxidation of lipids, signalling through Toll-like receptor (TLR) 4, and the production of TSLP that drives Th2 responses following subcutaneous immunisation [42]. Because many allergens exhibit endogenous protease activity, it is therefore also possible that inhaled allergens might themselves contribute to enhanced expression of TSLP by AEC. 

To date, however, there are no reported in vitro studies using AEC isolated from children nor have there been studies on the role of TSLP in an animal model of childhood asthma.

Further support for a role for TSLP in the development of asthma comes from studies associating single nucleotide polymorphisms in the *TSLP *gene or its promoter region with an increased risk of developing asthma in childhood [43, 44].

### 2.2. Role of IL-25

Epithelial cell-derived IL-25 is increasingly recognised as being important in the induction of allergic inflammation [45]. TSLP-activated DC induce strong upregulation of the receptor for IL-25 on Th2 cells, thus linking these two cytokine pathways [46]. Recent studies have identified novel populations of cells involved in the innate host response, which contribute to maintaining and enhancing the Th2-biased response by secreting cytokines such as IL-5 and IL-13 in response to IL-25 [47].

We have shown that IL-25 produced by AEC plays a key role in the induction of a Th2-biased inflammatory response following respiratory viral infection [35]. Furthermore, we have recently provided convincing evidence that IL-25 is of crucial importance in the induction phase of childhood asthma. For the latter studies, we used a novel animal model of neonatal infection with pneumonia virus of mice (PVM), a species-specific paramyxovirus which simulates RSV infection in human infants [48]. Following subsequent intranasal sensitisation with ovalbumin and long-term low-level challenge, these mice developed inflammation and remodelling typical of chronic asthma, together with a Th2-biased immunological response [49]. By themselves, neither infection nor allergen exposure led to the development of an asthmatic phenotype. This model therefore simulates the interaction between early childhood infection with RSV and sensitisation to inhaled allergens during the development of childhood asthma. 

In this model, we found that there was significant upregulation of expression of IL-25 following neonatal infection with PVM. Therefore, we tested the effects of administration of a neutralising antibody to IL-25 on the development of the asthmatic phenotype, in comparison to administration of an antibody to IL-4, which has long been recognised as having a crucial role in the induction of Th2 responses [50]. Anti-IL-25, administered either during chronic challenge or in early life alone, prevented key changes of airway remodelling such as subepithelial fibrosis and epithelial hypertrophy, and suppressed development of a Th2 response. Anti-IL-4 was more effective in inhibiting allergic inflammation, prevented goblet cell change but not other features of remodelling, and also suppressed development of a Th2 response [51]. These novel findings suggest that blocking induction of a Th2 response during the neonatal period or later in childhood could be effective for primary prevention of asthma, and that IL-25 might play a crucial role in this process.

### 2.3. Role of Other Factors

GM-CSF clearly plays a key role in the development of DC [52] and differentiated human respiratory epithelial cells release significant amounts of GM-CSF when exposed to DEP in vitro [53]. However, there is currently no direct evidence of a role for GM-CSF produced by AEC during the induction phase of childhood asthma.

There has been much speculation about the possible contribution of IL-33 to the development of asthma, given the capacity of this cytokine to promote DC maturation towards a Th2-inducing phenotype in vitro [54]. While IL-33 is expressed by airway epithelium in asthmatics [55], there is once again a paucity of direct evidence for a role in the induction phase of allergic asthma.

## 3. Triggering Cytokine Release by Airway Epithelial Cells

There is now considerable evidence that an important mechanism by which environmental irritants cause injury to AEC is via inducing the production of reactive oxygen species (ROS). This has been well studied in vitro in response to injury by RSV [56, 57]. Furthermore, activation of antioxidant defence mechanisms, through binding of the transcription factor Nrf2 to antioxidant response elements (AREs), has been shown to be an important part of the host response to RSV infection in vivo. RSV infection caused induction of various ARE-driven enzymes, and gene-targeted mice deficient in Nrf2 had significantly lower levels of reduced glutathione in the lungs, higher levels of oxidative modification of lung proteins and lipids, and developed significantly more severe RSV disease, both in terms of inflammation and epithelial injury [58]. Similarly, in vitro studies have demonstrated that DEP induce oxidative stress, with greater effects on AEC than on other target cells such as pulmonary macrophages [59]. There is clear evidence that paracetamol (acetaminophen) decreases intracellular levels of reduced glutathione and thus predisposes to injury by ROS [60].

Oxidative stress leads to activation of key intracellular signalling mechanisms, for example, the mitogen-activated protein kinase (MAPK) and NF-*κ*B pathways, and these can be inhibited by administration of antioxidants [61, 62]. Activation in turn leads to the generation of chemoattractants and proinflammatory cytokines. Whether similar events occur in vivo during the induction phase of paediatric asthma has not been formally demonstrated. However, the concept that oxidant injury may be a key early event is supported by evidence that functional polymorphisms in oxidant defence genes increase the risk of developing asthma in childhood [63, 64].

Environmental irritants may also trigger AEC via pattern recognition receptors, including the predominantly cell surface or endosomal TLRs and the cytoplasmic Nod-like receptors (NLRs). These receptors are key components of host innate defences, capable of recognising conserved molecular patterns associated with pathogens or with cellular damage [65]. TLRs signal via adaptor proteins, notably MyD88, leading to activation of MAPK and NF-*κ*B signalling pathways [66]. Whether TLR-dependent signalling promotes the development of a Th2-biased response following exposure to viral infection or environmental irritants is not altogether clear. However, the potential importance of TLR-mediated responses is supported by evidence that polymorphisms in genes for TLR2 and TLR4 are related to increased prevalence of asthma from birth up to the age of 8 years [67]. Members of the NLR family assemble into large multiprotein complexes, termed inflammasomes, which activate caspase-1, a proteolytic enzyme that cleaves and thus activates cytokines such as IL-1*β* and IL-18 for secretion [65]. Expression of NLRs has been demonstrated in the airway epithelium [68], but their role in the response to injury by environmental irritants, or in driving allergic inflammation, has not hitherto been investigated.

Another potentially important mechanism by which cytokine release from AEC might be triggered is via the enzymatic activity of some allergens on protease-activated receptors. For example, fungal antigens appear to be able to elicit secretion of TSLP via PAR-2 activation [69].

## 4. A Proasthmatic Epithelial Phenotype

An intriguing question, especially in relation to early-life viral infection, is whether environmental injury might lead to induction of a relatively stable epithelial phenotype that characterises and/or promotes the development of asthma [70]. There is no doubt that the airway epithelium of asthmatics is different to that of nonasthmatics, with evidence of abnormal proliferation/repair and enhanced production of proinflammatory cytokines [71–73]. Notably, this includes enhanced expression of TSLP and GM-CSF by asthmatic AEC [74, 75]. Furthermore, impaired production of interferons has been related to the increased susceptibility of asthmatics to viral infections [76, 77]. However, what is not clear is whether this is a reason for development of asthma, or an effect of a predisposing factor or of asthma itself. The issue is relevant to the ongoing debate about the relationship between viral infection and asthma [78]. Interestingly, recent evidence suggests that in a Th2-biased environment, which induces IL-13-driven mucous cell change in the airway epithelium, the altered epithelial lining may be more susceptible to infection by rhinovirus [79]. Thus, the mucous cell hyperplasia and other remodelling of the airway epithelium associated with the development of childhood asthma [80] may predispose to progression of the asthmatic phenotype, by promoting a vicious cycle of viral and allergic inflammation.

## 5. Conclusion

Injury to AEC by environmental factors may have an important role in initiating a cascade of responses that can lead to the development of asthma in childhood. Oxidant stress by environmental injurious agents may activate a variety of intracellular signalling pathways, that in turn drive the synthesis and secretion of multiple cytokines able to promote the recruitment of T cells and their polarisation towards a Th2 cytokine-secreting phenotype. However, at present there are many gaps in our understanding of the pathogenetic sequence of events. Further studies in animal models of childhood asthma may help to identify interventions that could reduce the risk of allergic asthma. Caution is warranted, however, because the mechanisms are likely to be complex and targeting single cytokines or regulatory pathways is rarely successful in asthma [81, 82].
